# Risk Allele rs117026326-Mediated Alternative Splicing of GTF2I Promotes B Cell Proliferation in Primary Sjögren's Syndrome

**DOI:** 10.1155/jimr/4821639

**Published:** 2025-02-18

**Authors:** Chaowen Luo, Chaofeng Lian, Jinlei Sun, Liling Zhao, Shuo Zhang, Yongzhe Li, Hua Chen, Fengchun Zhang

**Affiliations:** ^1^Department of Rheumatology and Clinical Immunology, Peking Union Medical College Hospital, Chinese Academy of Medical Sciences and Peking Union Medical College, Beijing, China; ^2^Department of Rheumatology and Clinical Immunology, The First Affiliated Hospital of Zhengzhou University, Zhengzhou, China; ^3^Department of Clinical Laboratory, Peking Union Medical College Hospital, Chinese Academy of Medical Sciences and Peking Union Medical College, Beijing, China; ^4^Key Laboratory of Rheumatology and Clinical Immunology, Ministry of Education, Beijing, China

**Keywords:** B cell, GTF2I, primary Sjögren's syndrome, rs117026326

## Abstract

**Objectives:** Primary Sjögren's syndrome (pSS) is associated with a risk allele T of rs117026326 located at a potential splicing enhancer within the intronic region of general transcription factor II-I (GTF2I). This study aimed to explore the rs117026326-regulated alternative splicing of GTF2I and its role in B cell overactivation in pSS.

**Methods:** GTF2I isoform expressions and rs117026326 genotypes of pSS peripheral blood mononuclear cells (PBMCs) were examined using quantitative PCR and Sanger sequencing, respectively. GTF2IΔ was overexpressed in B cells, T cells, and macrophages using plasmid transfection. Proliferation of B cells and T cells was determined using Cell Counting Kit-8 (CCK8) assay. CD4^+^ T cell differentiation was inspected using flow cytometry. Proinflammatory cytokine production of macrophages was investigated using quantitative PCR. c-FOS expression in GTF2IΔ-transfected B cells was tested by quantitative PCR, and proliferation of GTF2IΔ-transfected B cells treated with c-FOS siRNA or c-FOS inhibitor was interrogated using CCK8 assay.

**Results:** pSS patients with risk allele of rs117026326 expressed higher levels of GTF2IΔ and GTF2Iζ isoforms. GTF2IΔ expression was correlated with serum immunoglobulin G (IgG). GTF2IΔ promoted B cell proliferation and upregulated c-FOS expression. Knocking down or inhibition of c-FOS reversed B cell proliferation driven by GTF2IΔ.

**Conclusion:** pSS risk allele of rs117026326 modulates alternative splicing of GTF2I and upregulates GTF2IΔ isoform, which promotes B cell proliferation through enhancing binding and transcription of c-FOS.

## 1. Introduction

Primary Sjögren's syndrome (pSS) is a systemic autoimmune disease characterized by B cell activation. pSS patients exhibit biomarkers of B cell activation, including polyclonal hypergammaglobulinemia, elevated levels of free light chains, and autoantibodies such as rheumatoid factor (RF), anti-Ro/anti-Sjögren's syndrome A antigen (anti-SSA) antibodies, and anti-La/SSB antibodies. Moreover, B cell infiltration into exocrine glands and frequent formation of ectopic germinal centers (GCs). Additionally, the risk of B cell lymphoma is dramatically increased in pSS patients. Thus, B cell is a major therapeutic target for pSS [1, 2].

Genome-wide association studies (GWAS) unveil many single-nucleotide polymorphisms (SNPs) associated with pSS [3, 4]. Among them, rs117026326 is a novel SNP associated with Han Chinese pSS patients first discovered in our GWAS [4], which is confirmed by another GWAS in Chinese pSS patients [5]. Moreover, rs117026326 is associated only with anti-SSA-positive pSS but not anti-SSA-negative pSS [6]. Furthermore, rs117026326 is associated with other autoimmune diseases in East Asian populations, including rheumatoid arthritis (RA) [7], systemic lupus erythematosus (SLE) [8], systemic sclerosis (SSc) [9, 10], and primary biliary cholangitis (PBC) [11]. These data indicate that rs117026326 potentially regulates the immune response of pSS and related autoimmune diseases, whose mechanism remains elusive.

rs117026326 is located within the intronic region of general transcription factor II-I (GTF2I) at 7q11.23, which is a transcription factor binding to promoter Initiator (Inr) elements and E-box elements. To delineate the functional role of rs117026326, we examined its location and performed expression quantitative trait loci (eQTL) analysis. Although GTF2I is highly expressed in salivary gland cells of pSS patients with risk allele of rs117026326 [12], eQTL analysis did not find a correlation between genotype at rs117026326 and GTF2I expression on gene expression data of lymphoblastoid cell lines [4]. GTF2I comprises 36 exons and produces six alternatively spliced isoforms. rs117026326 is located at a potential splicing enhancer, which might modulate the binding of splicing factors in its splicing process and regulate GTF2I isoforms.

In this study, we examined the GTF2I isoforms in different alleles of rs117026326. We further explored the phenotypes of B cells, T cells, and macrophages transfected with GTF2I isoforms. Finally, we investigated the mechanism of GTF2I isoform-induced B cell proliferation.

## 2. Methods

### 2.1. Subjects

pSS patients who met the 2002 American–European Consensus Group (AECG) classification criteria for pSS and sex- and age-matched healthy controls (HCs) were enrolled from Peking Union Medical College Hospital between December 2015 and October 2016. All patients did not receive glucocorticoids or immunosuppressants for over 3 months before enrollment. The demographic and clinical characteristics of patients are listed in Supporting Information 13: Table S1.

### 2.2. Genotyping

Genomic DNA was extracted using the phenol–chloroform method and was genotyped for rs117026326 by Sanger sequencing.

### 2.3. Quantitative RT-PCR

Total RNA was collected with RNA-Quick Purification Kit (Yishan Biotechnology) and was quantified using NanoDrop 2000c spectrophotometer (NanoDrop). cDNA was synthesized with PrimeScript RT Master Mix (Takara), and real-time PCR was performed with TB Green Premix Ex Taq II (Takara) using Roche 480 II thermocycler. Gene relative expression was calculated using the comparative ΔΔCT method with GAPDH as internal control. Primer sequences are listed in Supporting Information 13: Table S3.

### 2.4. Cells

Raji and Ramos B cell lines, Jurkat T lymphoblast cell line, and THP1 leukemia monocytic cell line were obtained from Procell. Peripheral blood mononuclear cells (PBMCs) were isolated using Ficoll-Paque gradient centrifugation (Dakewe). CD4^+^CD45RA^+^CD45RO^−^CCR7^+^CD25^−^ naive CD4^+^ T cells were purified from PBMCs using naïve CD4^+^ T Cell Isolation Kit II (Miltenyi Biotec), with a purity of over 97% by flow cytometry. Cells were incubated in RPMI-1640 medium (Gibco) supplemented with 10% fetal bovine serum (FBS) (Gibco) at 37°C and 5% CO_2_ for 3 days. Cell lines were transfected with pcDNA3.1(+)-GTF2IΔ plasmid (100 ng, OBiO Technology) using Lipofectamine 3000 (Invitrogen) or c-FOS siRNA (100 nM, Supporing Information 13: Table S4, GenePharma) using Lipofectamine RNAiMAX (Invitrogen). Naïve CD4^+^ T cells were electroporated with GTF2IΔ plasmid (100 ng, OBiO Technology) using 4D-Nucleofector System (Lonza). THP1 cells were induced by PMA (50 ng/mL, Yishan Biotechnology) for 24 h and then were stimulated by LPS (20 ng/mL, Beyotime) for 4 h. For c-FOS gene expression studies, cells were starved in serum-free RPMI-1640 medium overnight and then were stimulated in RPMI-1640 medium supplemented with 20% FBS for 30 min. Cells were treated with T-5224 (50 µM, Selleck) or dimethyl sulfoxide (DMSO) (Solarbio) and were incubated in RPMI-1640 medium supplemented with 10% FBS at 37°C and 5% CO_2_ for 3 days.

### 2.5. Proliferation Assay

Cells (25,000/100 µL) were incubated for 3 days before Cell Counting Kit-8 (CCK8, 10 µL, Aoqing Biotechnology) was added for 4–8 h. Optical density (OD) was measured by absorbance at 450 nm using Multiskan FC Microplate Reader (Thermo Fisher).

### 2.6. Naive CD4^+^ T Cell Activation and Differentiation

Naive CD4^+^ T cells were activated by immobilized anti-CD3 antibody (5 µg/mL, BD Bioscience) and soluble anti-CD28 antibody (5 µg/mL, BD Bioscience) in X-VIVO medium (Lonza) supplemented with 5% FBS (Gibco) at 37°C, 5% CO_2_ for 5 days.

### 2.7. Flow Cytometry

CD4 (RPA-T4), CD25 (BC96), CXCR5 (J252D4), PD-1 (EH12.2H7), IFNγ (4S.B3), and IL-17A (BL168) from Biolegend and FOXP3 (PCH101) were purchased from eBioScienes. Cells were stained with antibodies (1:100) at 4°C in the dark for 30 min. For intracellular cytokine staining, cells were stimulated with a leukocyte activation cocktail (BD Bioscience) for 4 h and then fixed and permeabilized using Cytofix/Cytoperm (BD Bioscience) for 1 h, followed by staining with antibodies (1:100). For FOXP3 staining, cells were fixed and permeabilized using FOXP3 staining kit (eBioScienes). Data were acquired using BD Aria II Flow Cytometer (BD Bioscience) and were analyzed using FlowJo X (Tree Star).

### 2.8. Statistical Analysis

Genotyping of pSS patients and HCs was checked for Hardy–Weinberg equilibrium using chi-squared test. Categorical variables were described as number (percentage) and were compared with chi-squared test. Continuous variables were summarized as mean (standard deviation [SD]) or median (interquartile range [IQR]) according to Shapiro–Wilk test. Two groups were compared with *t*-test for normally distributed data or Mann–Whitney test for non-normally distributed data. Multiple groups were compared using one-way analysis of variance followed by Dunnett's test for normally distributed data or Kruskal–Wallis test for non-normally distributed data. The association of variables with GTF2IΔ expression was assessed using Spearman's correlation test for non-normally distributed data. A two-sided value of *p* < 0.05 was considered statistically significant. All analyses were conducted using Prism v. 7.0 (GraphPad) or SPSS v. 25.0 (IBM).

## 3. Results

### 3.1. Elevated Expression of GTF2IΔ in pSS Patients With Risk Allele of rs117026326

**Figure 1:**
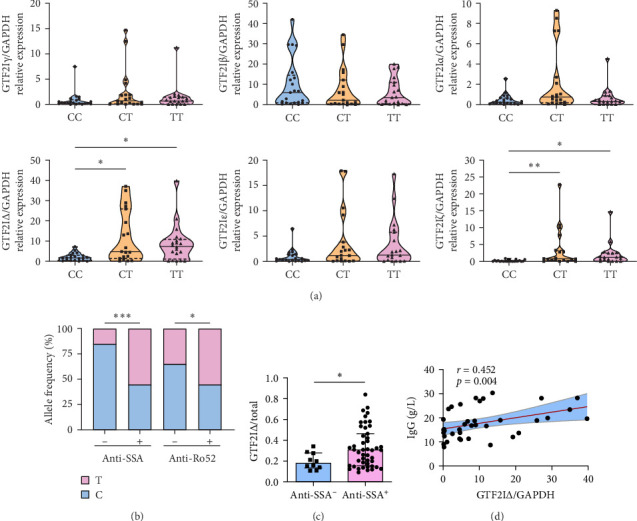
Elevated expression of GTF2IΔ isoform in pSS patients with rs117026326 risk allele. (A) Relative expressions of general transcription factor II-I (GTF2I) isoforms in peripheral blood mononuclear cell (PBMC) from pSS patients with CC (*n* = 21), CT (*n* = 19), or TT (*n* = 19) genotypes at rs117026326. (B) rs117026326 allele frequency in pSS patients with anti-SSA and anti-Ro52. (C) Abundance of GTF2IΔ isoforms in pSS patients with anti-SSA. (D) Correlation analysis of serum IgG and GTF2IΔ in pSS patients with risk allele T at rs117026326. Data are shown as median with interquartile range. *⁣*^*∗*^*p*  < 0.05, *⁣*^*∗∗*^*p*  < 0.01, and *⁣*^*∗∗∗*^*p*  < 0.001 by Mann–Whitney test and Spearman's correlation test for non-normal distribution. Anti-Ro52, anti-Ro52 antigen; anti-SSA, anti-Sjögren's syndrome A antigen; IgG, immunoglobulin G; pSS, primary Sjögren's syndrome.

We first confirmed that the frequency of rs117026326 risk allele T was higher in pSS (*n* = 196) than HCs (*n* = 180) using Sanger sequencing (Supporting Information 13: Table S2, Supporting Information 1: Figure S1). The expression of total GTF2I was comparable among genotypes (Supporting nformation 2: Figure S2). The risk allele T of rs117026326 potentially abrogates the exon splicing enhancer of splicing factor2/alternative splicing factor (SF2/ASF) using ESE finder 3.0 (Supporting Information 3: Figure S3). To address whether GTF2I alternatively spliced transcripts were affected by risk allele T, we examined relative expressions of six GTF2I isoforms in PBMCs from pSS patients with CC (*n* = 21), CT (*n* = 19), or TT (*n* = 19) genotypes of rs117026326 using quantitative PCR whose products were verified by Sanger sequencing (Figures S4–S5). Interestingly, the risk allele T of rs117026326 was significantly associated with an increased abundance of GTF2IΔ and GTF2Iζ (Figure 1A). Since GTF2IΔ is one of the two most abundant isoforms in PBMCs, we focused on this isoform (Supporting Information 6: Figure S6). The risk allele T frequencies were higher in pSS patients with anti-SSA or anti-Ro52 antibodies than those without anti-SSA or anti-Ro52 antibodies (Figure 1B). Additionally, the abundance of GTF2IΔ was significantly higher in pSS patients with anti-SSA antibody (Figure 1C) or pulmonary involvement (Supporting Information 7: Figure S7). Furthermore, GTF2IΔ expression was positively correlated with serum immunoglobulin G (IgG) (*r* = 0.452, *p*=0.004) in pSS patients with the risk allele T (Figure 1D). Collectively, these data confirmed that risk allele T of rs117026326 was associated with GTF2IΔ in pSS patients.

### 3.2. GTF2IΔ Promotes B Cell Proliferation

**Figure 2:**
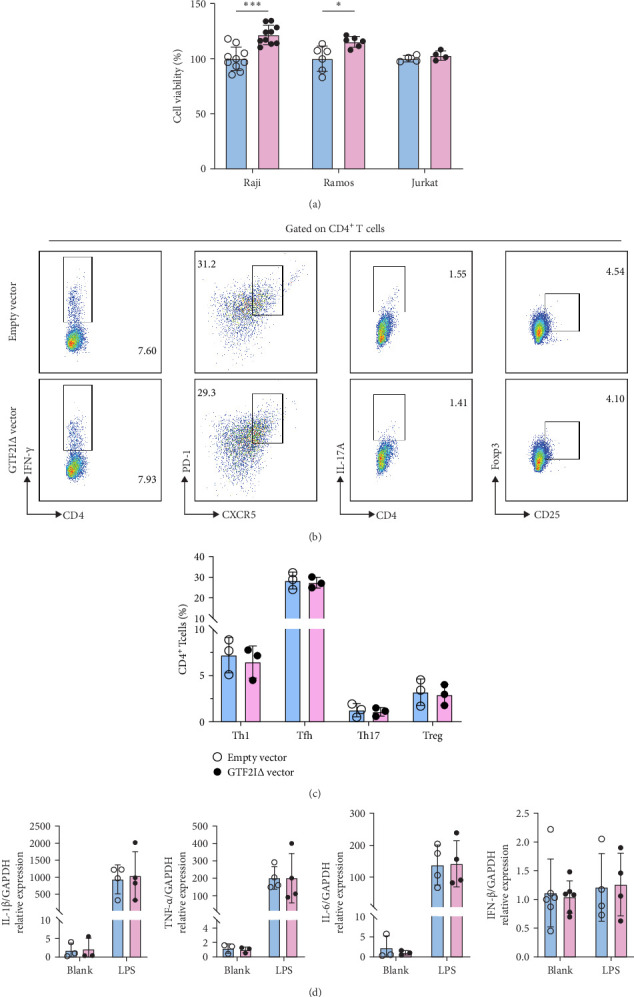
GTF2IΔ isoform promotes B cell proliferation. (A) Proliferation of Raji, Ramos, and Jurkat cells transfected with GTF2IΔ vector on day 3 by CCK8 assay. (B, C) IFN-γ^+^ Th1, CXCR5^+^PD-1^+^ Tfh, IL-17A^+^ Th17, and CD25^hi^Foxp3^+^ Treg differentiation by naive CD4^+^ T cells (*n* = 3) transfected with GTF2IΔ vector on day 5. (D) IL-1β, TNF-α, IL-6, and IFN-β relative expression of LPS-elicited THP1 cells transfected with GTF2IΔ vector on day 3. Data are shown as mean ± SD and were obtained from three independent experiments. *⁣*^*∗*^*p*  < 0.05 and *⁣*^*∗∗∗*^*p*  < 0.001 by Student's *t*-test. LPS, lipopolysaccharide.

Given that GTF2IΔ was elevated in pSS PBMCs, we examined whether GTF2IΔ regulated B cell, T cell, or macrophage dysregulation in pSS. We overexpressed GTF2IΔ in B cell, T cell, and macrophage cell lines (Supporting Information 8: Figure S8). GTF2IΔ significantly promoted Raji and Ramos B cell proliferation (Figure 2A) and modestly induced B cell apoptosis (Supportin Information 9: Figure S9). For T cells, GTF2IΔ did not promote proliferation (Figure 2A), IFN-γ^+^ Th1, CXCR5^+^PD-1^+^ Tfh, IL-17 A^+^ Th17, or CD25^hi^Foxp3^+^ Treg differentiation (Figure 2b,c, Supporting Information 10: Figure S10). For macrophages, GTF2IΔ did not promote IL-1β, TNF-α, IL-6, and IFN-β production (Figure 2D). Together, these data indicated that elevated GTF2IΔ in pSS promoted excessive B cell proliferation and contributed to B cell overactivation in pSS.

### 3.3. GTF2IΔ Upregulates c-FOS to Promote B Cell Proliferation

**Figure 3:**
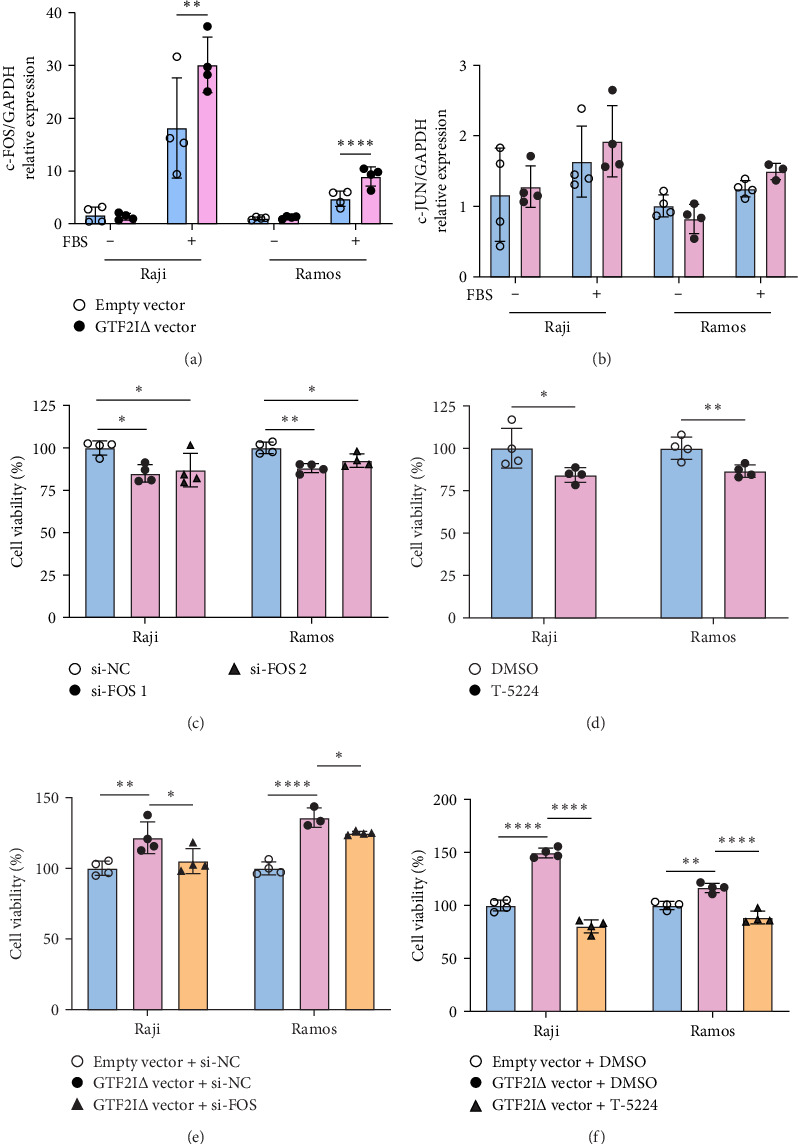
GTF2IΔ isoform upregulates c-FOS to promote B cell proliferation. (A) c-FOS and (B) c-JUN relative expressions of Raji and Ramos cells transfected with GTF2IΔ vector on day 3. Proliferation of Raji and Ramos transfected with (C) c-FOS siRNA or (D) T-5224 on day 3 by CCK8 assay. (E) Proliferation of Raji and Ramos transfected with GTF2IΔ vector and (or) c-FOS siRNA on day 3 by CCK8 assay. (F) Proliferation of Raji and Ramos treated with GTF2IΔ vector and (or) T-5224 on day 3 by CCK8 assay. Data are shown as mean ± SD and were obtained from three to five independent experiments. *⁣*^*∗*^*p*  < 0.05, *⁣*^*∗∗*^*p*  < 0.01, and *⁣*^*∗∗∗∗*^*p*  < 0.0001 by Student's *t*-test or ANOVA. DMSO, dimethyl sulfoxide; FBS, fetal bovine serum.

We further investigated the underlying mechanism of B cell proliferation mediated by GTF2IΔ. GTF2I binds c-FOS promoter and activates c-FOS transcription [13, 14], and a public GTF2I ChIP-seq dataset (GSE63057) of fibroblast cells [15] suggests that GTF2I binding peaks are located in FOS promoter (Supporting Information 11: Figure S11). Consistently, expression of c-FOS, but not c-JUN, was significantly upregulated in Raji and Ramos cells transfected with GTF2IΔ vector (Figure 3a,b). Furthermore, both c-FOS knockdown and c-FOS inhibitor, T-5224, attenuated B cell proliferation (Figure 3c,d). Finally, the proliferation of Raji and Ramos cells promoted by GTF2IΔ overexpression was largely abolished by c-FOS knockdown or T-5224 (Figure 3e,f). Therefore, these data implicated that GTF2IΔ promoted B cell proliferation through upregulating c-FOS.

## 4. Discussions

In this study, we first confirmed that rs117026326 risk allele is associated with pSS. Second, we demonstrated that GTF2IΔ expression was upgraded in pSS patients with rs117026326 risk allele. Third, GTF2IΔ promoted B cell proliferation. Finally, GTF2IΔ upregulated c-FOS expression, which subsequently promoted B cell proliferation (Supporting Information 12: Figure S12). Overall, our study indicated that rs117026326 risk allele played a role in B cell overactivation in pSS.

B cell overactivation is a hallmark of pSS, which is stimulated by environmental triggers in the presence of genetic and epigenetic dysregulation [16]. Virus infections directly activate B cells via pattern recognition receptors (PRRs) such as Toll-like receptors (TLRs) and retinoic acid-inducible gene-I (RIG-I)-like receptors (RLRs), through type I interferons (IFNs) and proinflammatory cytokines. IFNs promote myeloid cells and salivary gland epithelial cells (SGECs) to overproduce B cell-activating factor (BAFF) and crosstalk with B cells to instruct B cell activation [17, 18]. Risk alleles in B lymphocyte kinase (BLK), C-X-C chemokine receptor type 5 (CXCR5), and PR domain 1 (PRDM1) are identified in GWAS, which regulates B cell receptor (BCR) signaling, GC organization, and plasma cell activation, respectively. Upregulated epithelial–mesenchymal interacting protein 1 (EPSTI1) in pSS promotes B cell activation through NF-κB signaling [19]. Our study revealed that risk allele of rs117026326 was associated with anti-SSA antibodies and upregulated GTF2IΔ expression in pSS, which enhanced B cell proliferation. Therefore, our study provided a new mechanism for the pSS-risk allele of rs117026326 in B cell overactivation of pSS. For pSS patients carrying T allele of rs117026326, GTF2IΔ is overexpressed and promotes B cell proliferation. Targeting GTF2IΔ might be a therapeutic approach for alleviating B cell overactivation in pSS.

We demonstrated that GTF2IΔ promoted B cell proliferation. GTF2I is a multifunctional transcription factor modulating transcription that is phosphorylated in response to B and T cell receptor signaling pathways, which modulates B cell and T cell responses [20]. GTF2I is tumorigenic, enhancing cell proliferation of B cell lymphoma and lymphoblastic leukemia [21–24]. In B cells, BTK-mediated GTF2I phosphorylation activates BCR signaling [25], which is impaired in GTF2I-silenced murine B cells [26]. GTF2I-deficient murine B cells show higher proliferation capacities [27], and GTF2I-silenced murine B cells present NF-κB signaling activation [26]. GTF2I also induces immunoglobulin heavy-chain transcription by interacting with B cell-specific coactivator OCA-B and B cell transcription factor Bright [28–30]. In T cells, GTF2I interacts with inducible tyrosine kinase (Itk) [31] and activates the TCR-derived Vβ promoter [32, 33], although we did not observe modulation of T cell proliferation or differentiation by GTF2IΔ. Conversely, GTF2I activates NF-κB signaling by binding to p65 [12]. However, we did not find macrophage activation promoted by GTF2IΔ. GTF2I binds to c-FOS promoter and regulates its transcription in response to BCR, TCR, and growth factor signaling [13, 25, 31]. GTF2IΔ is stronger than the other isoforms in transcriptional activation of c-FOS [14], and GTF2Iβ represses c-FOS expression [34]. The regulation of the overall function of GTF2I is largely unknown. The aforementioned conflicting results might be dependent on the ratio between GTF2IΔ and GTF2Iβ, and total GTF2I deficiency may overshadow the partial defect induced by isoform silencing. Thus, GTF2I might regulate immune response differently in human and murine B cells and T cells, and further studies in human immune cells are warranted to understand the role of GTF2I in the immune response.

Alternative splicing (AS) is a vital transcriptional regulatory mechanism producing multiple mRNA with a single gene, which greatly expands transcriptome and proteome diversity. Aberrant AS plays essential roles in autoimmune disease [35, 36], with 60% of genes showing frequent alternatively spliced isoforms in T cells and B cells [37]. Noncoding SNPs l may disrupt splicing sites or create novel alternative binding sites, thereby influencing the efficiency or selection mode of alternative splicing. Risk allele of rs10774671 shifts pSS B cell splicing of OAS1 from p46 isoform to p42, p48, and p44 alternative transcripts, which are less responsive to IFN stimulation [38]. The protective CD72 genotype promotes exon 8 skipping and upregulates CD72Δex8 isoform in lupus B cells, which augments B cell apoptosis and downregulates immunoglobulin level [39]. PTPN22.6, a novel alternative splice form of PTPN22, is highly expressed in active RA, which leads to hyperactivation of T cells [40]. Type 1 diabetes-associated CTLA4 risk allele decreased sCTLA-4 isoform relative to the full-length isoform, which impairs the suppressive activity of Treg cells [41]. The expression of FOXP3 exon 2 splicing alleles is modulated by glycolysis and controls the Treg induction in multiple sclerosis and type 1 diabetes [42]. In this study, we proposed the splicing of GTF2I played a role in B cell proliferation, which expanded the scope of AS-regulated immune response and potentially implicated in other autoimmune diseases such as RA [7] and SLE [8].

AS modulation has been applied in autoimmune diseases. Exon-skipping therapy modulating BAFF mRNA splicing produces an alternative splice isoform ΔBAFF as a BAFF inhibitor, which attenuates B cell and plasma cell infiltration in the salivary gland and increases salivary flow in the NOD model [43]. Splice-switching oligonucleotides (SSOs) promote exon 7 skipping in TNFR2 mRNA and upregulate soluble TNFR2 isoform, which blocks TNF signaling and reduces inflammation in the arthritis model [44]. Therefore, targeting GTF2I splicing might be a therapeutic approach for pSS.

Our study has limitations. First, we observed enhanced B cell proliferation in B cell lines but not primary B cells, although our data were replicated in two different B cell lines. Second, we focused on the functional mechanisms of major isoform GTF2IΔ, and the potential role of other isoforms is worth further investigation. Third, we measured GTF2IΔ expression in pSS PBMCs, and whether GTF2IΔ expression in pSS B cells with risk allele of rs117026326 is elevated remains unclear. However, we tested the functional role of GTF2IΔ in T cells and B cells, the major composition of PBMCs, which probably expressed higher GTF2IΔ in patients with risk allele of rs117026326.

In summary, we demonstrate that the risk allele at rs117026326 modulates alternative splicing of GTF2I and upregulates GTF2IΔ isoform, which promotes B cell proliferation through enhancing binding and transcription of c-FOS. Our study uncovers the mechanism of rs117026326 association of pSS.

## Data Availability

The data from the current study are available from the corresponding author upon reasonable request.

## Supporting Information

Supporting Information 1 Figure S1: Representative Sanger sequencing of rs117026326 genotyping.

Supporting Information 2 Figure S2: Total GTF2I relative expressions in PBMCs from pSS patients.

Supporting Information 3 Figure S3: ESE finder prediction of rs117026326 in exon splicing enhancers.

Supporting Information 4 Figure S4: Schematics of GTF2I isoforms and rs117026326.

Supporting Information 5 Figure S5: Primer design and sequencing of GTF2I isoforms. (A) Schematics of GTF2I isoforms primer design. (B) Sanger sequencing of PCR products of GTF2I isoforms using primers of (A).

Supporting Information 6 Figure S6: Relative expressions of GTF2I isoforms in pSS PBMCs.

Supporting Information 7 Figure S7: GTF2IΔ expressions in pSS patients stratified by clinical presentations.

Supporting Information 8 Figure S8: Overexpression efficiency of GTF2IΔ vector.

Supporting Information 9 Figure S9: Apoptosis of Raji and Ramos cells transfected with GTF2IΔ vector.

Supporting Information 10 Figure S10: Gating strategy of Th1, Tfh, Th17, and Treg cells.

Supporting Information 11 Figure S11: ChIP-seq analysis of GTF2I (GSE63057).

Supporting Information 12 Figure S12: Schematic diagram of rs117026326 T risk allele-mediated B cell proliferation in pSS. rs117026326 T risk allele of pSS modulates alternative splicing of GTF2I and upregulates GTF2IΔ isoform expression, which binds and enhances c-FOS transcription, and ultimately promotes B cell proliferation. GTF2I, general transcription factor II-I; pSS, primary Sjogren's syndrome; SF2/ASF, splicing factor2/alternative splicing factor.

Supporting Information 13 Table S1: Clinical characteristics of pSS patients. Table S2: Allele and genotype frequencies of rs117026326 in pSS and HC. Table S3: PCR primers. Table S4: c-FOS siRNA sequences.
