# Supplementary figures and images for: Risk Allele rs117026326-Mediated Alternative Splicing of GTF2I Promotes B Cell Proliferation in Primary Sjögren's Syndrome
Source: J Immunol Res. 2025 Feb 18;2025:4821639. doi: 10.1155/jimr/4821639 (PMC11858827; doi:10.1155/jimr/4821639)

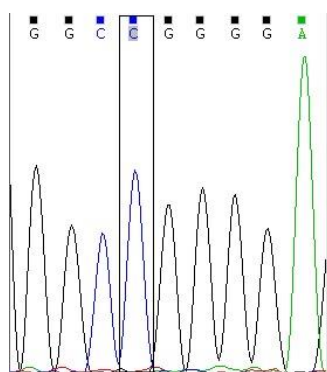

CC

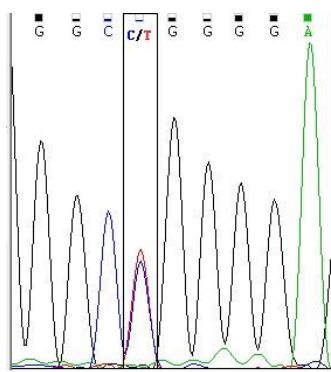

CT

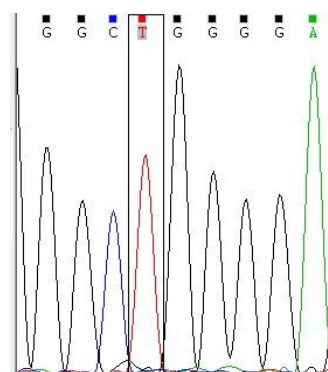

TT

Supplement: Supporting Information 1 — Figure S1: Representative Sanger sequencing of rs117026326 genotyping. [file 4821639.f1.pdf]

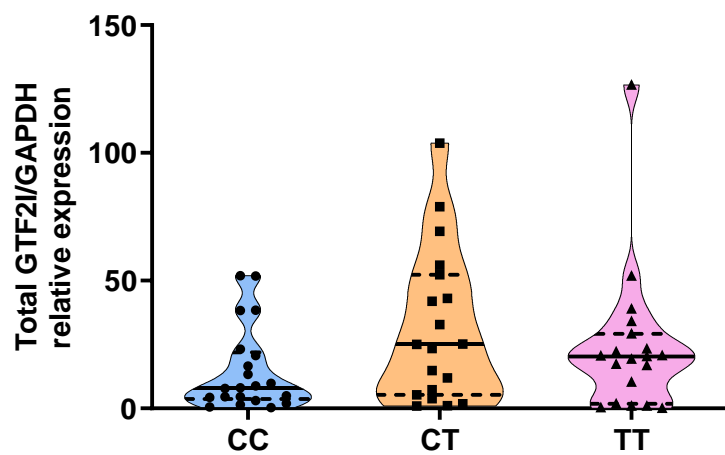

Supplement: Supporting Information 2 — Figure S2: Total GTF2I relative expressions in PBMCs from pSS patients. [file 4821639.f2.pdf]

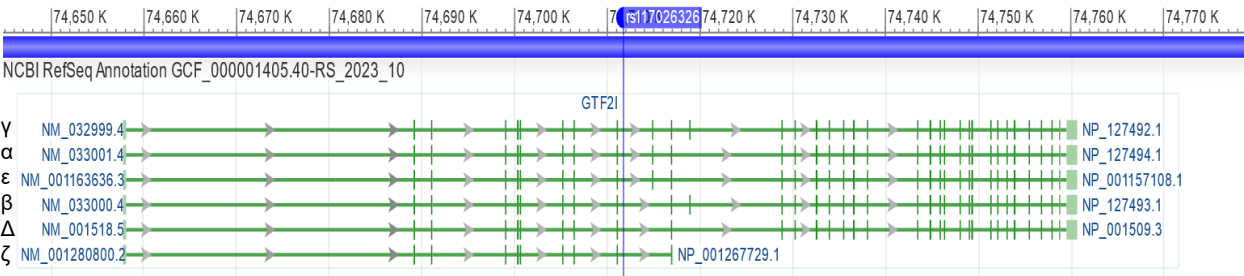

Supplement: Supporting Information 4 — Figure S4: Schematics of GTF2I isoforms and rs117026326. [file 4821639.f4.pdf]

A

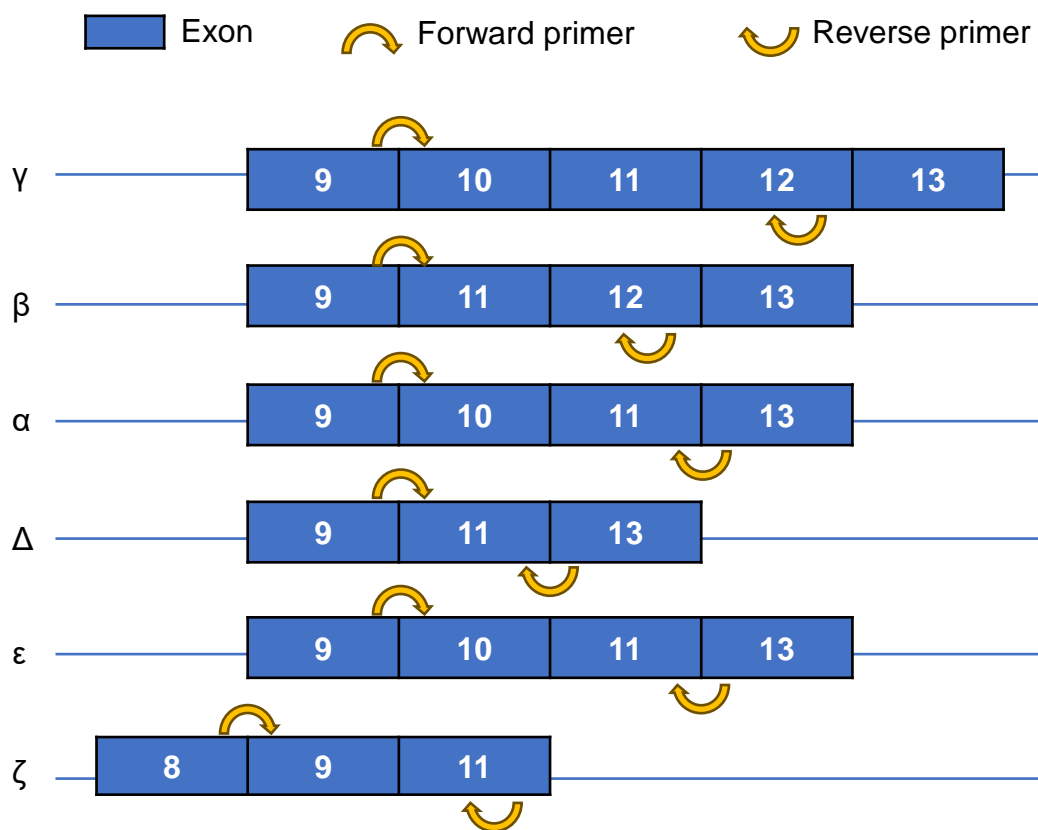

B

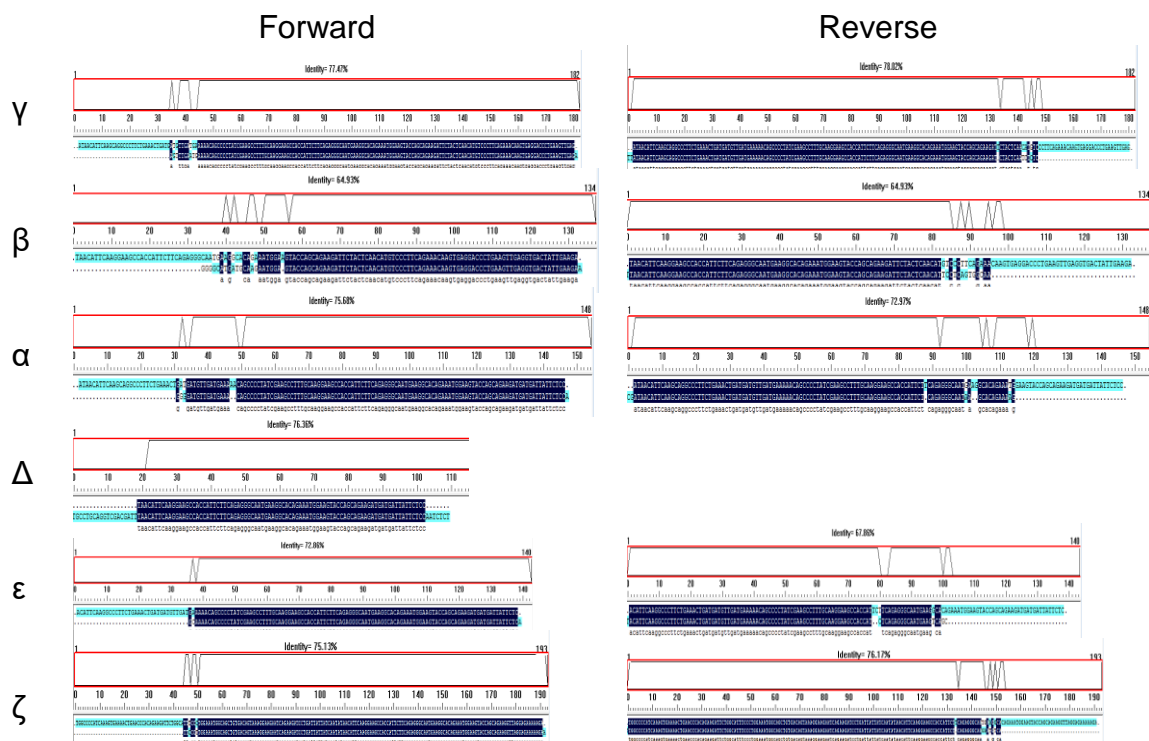

Supplement: Supporting Information 5 — Figure S5: Primer design and sequencing of GTF2I isoforms. (A) Schematics of GTF2I isoforms primer design. (B) Sanger sequencing of PCR products of GTF2I isoforms using primers of (A). [file 4821639.f5.pdf]

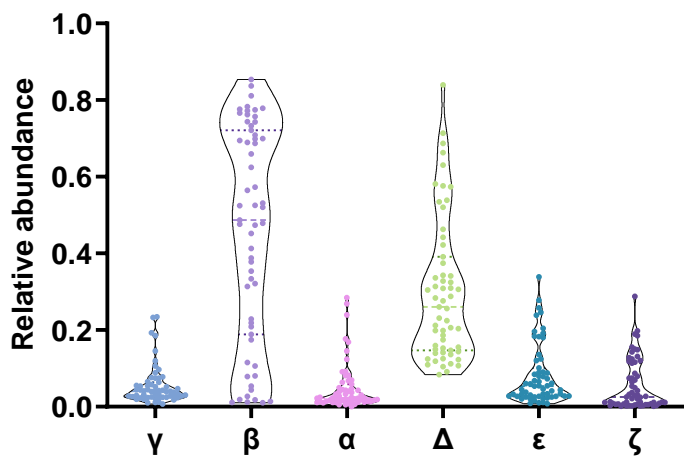

Supplement: Supporting Information 6 — Figure S6: Relative expressions of GTF2I isoforms in pSS PBMCs. [file 4821639.f6.pdf]

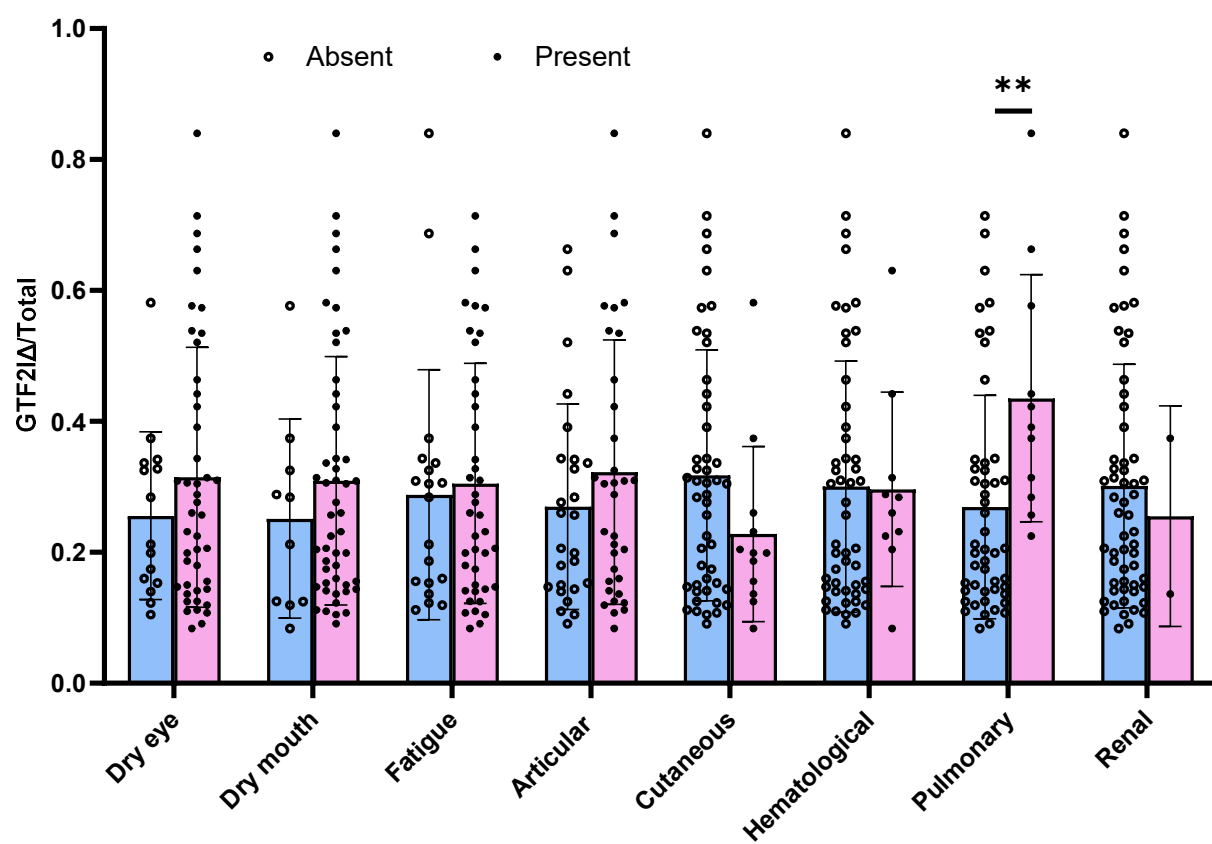

Supplement: Supporting Information 7 — Figure S7: GTF2IΔ expressions in pSS patients stratified by clinical presentations. [file 4821639.f7.pdf]

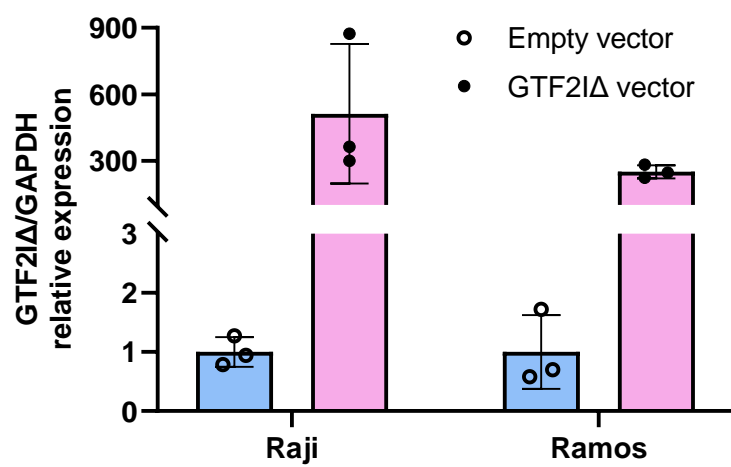

Supplement: Supporting Information 8 — Figure S8: Overexpression efficiency of GTF2IΔ vector. [file 4821639.f8.pdf]

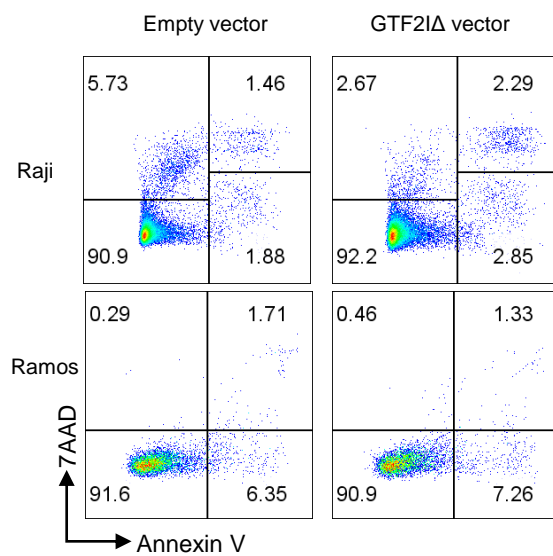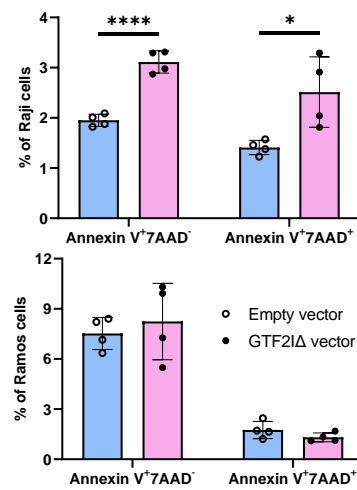

Supplement: Supporting Information 9 — Figure S9: Apoptosis of Raji and Ramos cells transfected with GTF2IΔ vector. [file 4821639.f9.pdf]

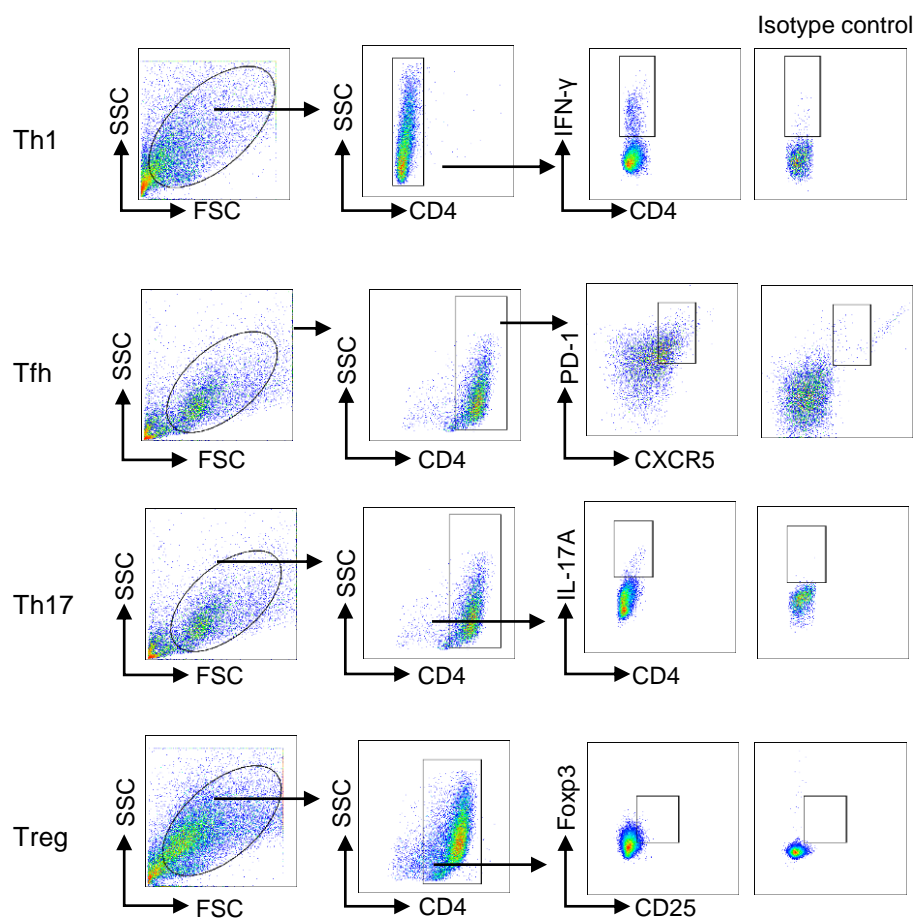

Supplement: Supporting Information 10 — Figure S10: Gating strategy of Th1, Tfh, Th17, and Treg cells. [file 4821639.f10.pdf]

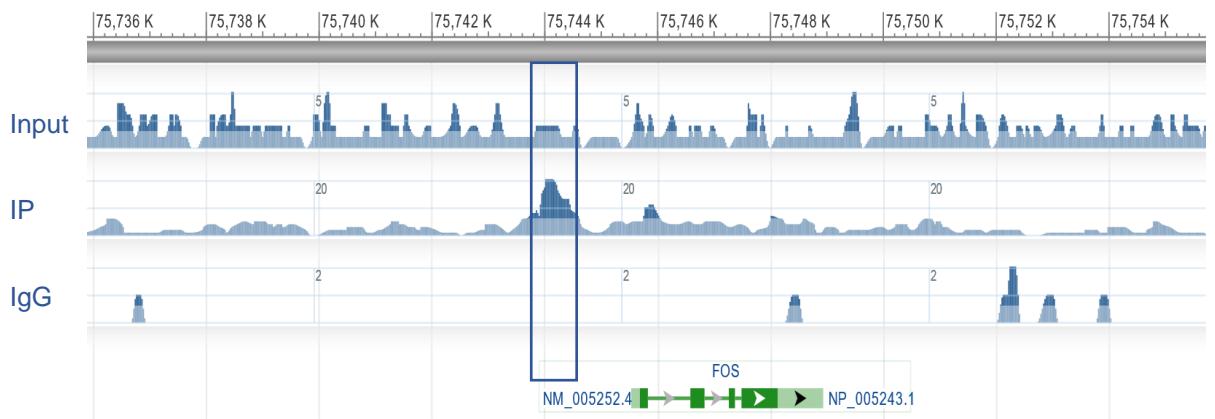

Supplement: Supporting Information 11 — Figure S11: ChIP-seq analysis of GTF2I (GSE63057). [file 4821639.f11.pdf]

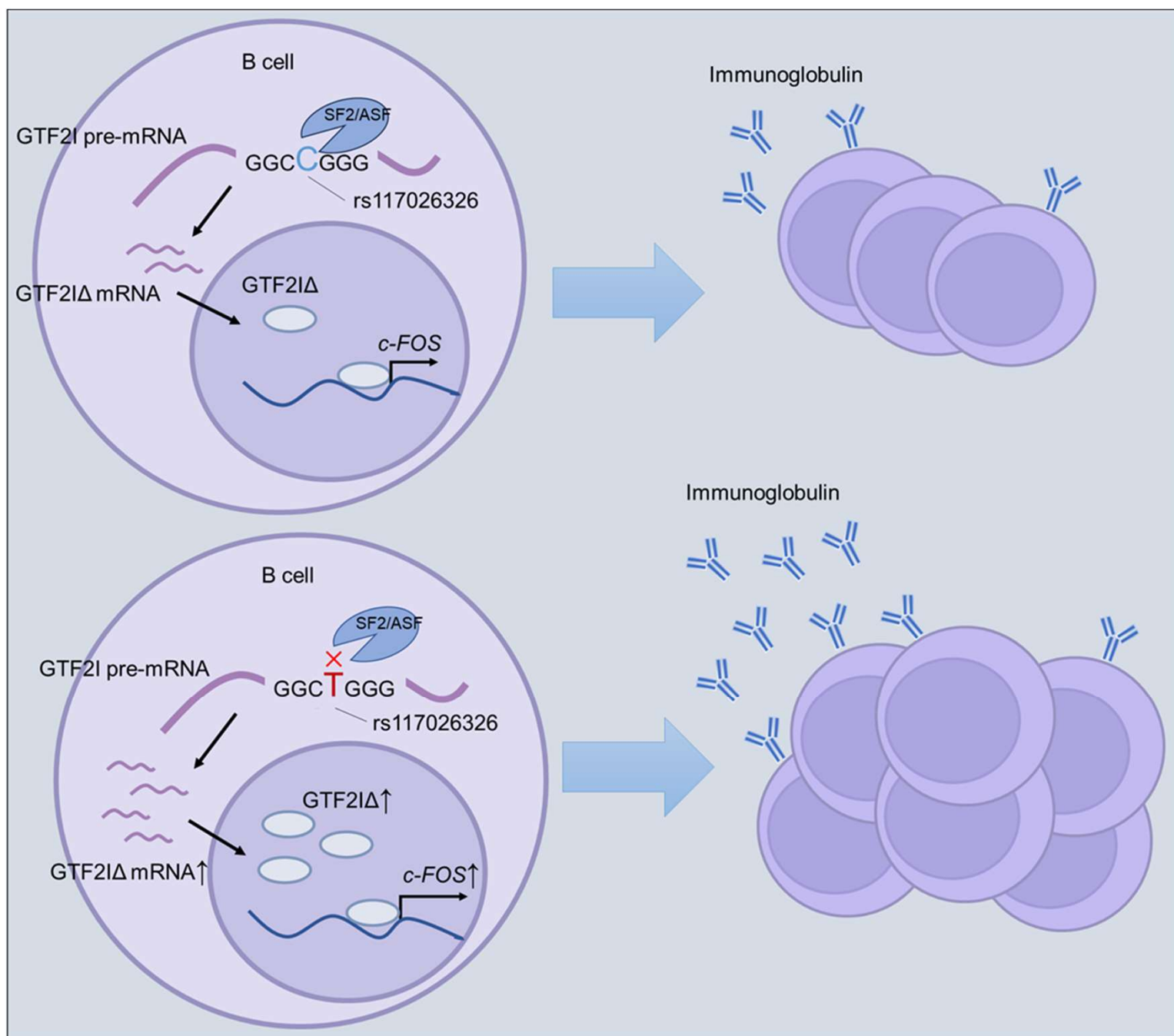

Supplement: Supporting Information 12 — Figure S12: Schematic diagram of rs117026326 T risk allele-mediated B cell proliferation in pSS. rs117026326 T risk allele of pSS modulates alternative splicing of GTF2I and upregulates GTF2IΔ isoform expression, which binds and enhances c-FOS transcription, and ultimately promotes B cell proliferation. GTF2I, general transcription factor II-I; pSS, primary Sjogren's syndrome; SF2/ASF, splicing factor2/alternative splicing factor. [file 4821639.f12.pdf]
