# Supplementary material for: Risk Allele rs117026326-Mediated Alternative Splicing of GTF2I Promotes B Cell Proliferation in Primary Sjögren's Syndrome
Source: J Immunol Res. 2025 Feb 18;2025:4821639. doi: 10.1155/jimr/4821639 (PMC11858827; doi:10.1155/jimr/4821639)
Supplement: Supporting Information 13 — Table S1: Clinical characteristics of pSS patients. Table S2: Allele and genotype frequencies of rs117026326 in pSS and HC. Table S3: PCR primers. Table S4: c-FOS siRNA sequences. [file 4821639.f13.docx]

**Supplementary Table S1.** Clinical characteristics of pSS patients

| **Variable** | **Patients (n=59)** |
| --- | --- |
| Female gender, n (%) | 58 (98) |
| Age, mean±SD, year | 48.6±12.6 |
| **Symptoms** |  |
| Dry eyes, n (%) | 44 (75) |
| Dry mouth, n (%) | 49 (83) |
| fatigue, n (%) | 40 (68) |
| ESSDAI domain |  |
| Articular domain, n (%) | 34 (58) |
| Cutaneous domain, n (%) | 13(22) |
| Hematological domain, n (%) | 10 (17) |
| Pulmonary domain, n (%) | 11 (19) |
| Renal domain, n (%) | 3 (5) |
| **Laboratory** |  |
| Positive ANA, n (%) | 59 (100) |
| Positive Anti-SSA antibody, n (%) | 49 (83) |
| Positive Anti-Ro52 antibody, n (%) | 39 (66) |
| Positive Anti-SSB antibody, n (%) | 22 (37) |
| IgG, median (first quartile, third quartile), g/L | 16.5 (11.9, 24.5) |
| RF, median (first quartile, third quartile), IU/mL | 22.9 (15.0, 65.4) |
| ESR, median (first quartile, third quartile), mm/h | 13.0 (8.0, 23.0) |

Quantitative data are expressed as mean with standard deviation (SD) for normal distribution and median (first quartile, third quartile) for non-normal distribution. Categorical variables are expressed as counts and percentages. ANA, Antinuclear antibody; Anti-SSA, Anti-Sjögren's syndrome A antigen; Anti-Ro52, Anti-Ro52 antigen; Anti-SSB, Anti-Sjögren's syndrome B antigen; ESR, erythrocyte sedimentation rate; IgG, Immunoglobulin G; pSS, primary Sjögren’s syndrome; RF, rheumatoid factor.

**Supplementary Table S2.** Allele and Genotype frequencies of rs117026326 in pSS and HC.

|  |  | **HC (n=180)** | **pSS (n=196)** | **OR (95%CI)** | ***p*-value** |
| --- | --- | --- | --- | --- | --- |
| Allele n (%) | C | 304 (84.4) | 279 (71.2) |  |  |
|  | T | 56 (15.6) | 113 (28.8) | 2.2(1.5-3.1) | <0.001 |
| Genotype n (%) | CC | 132 (73.3) | 104 (53.1) |  |  |
|  | CT | 40 (22.2) | 71 (36.2) |  |  |
|  | TT | 8 (4.5) | 21 (10.7) |  | <0.001 |

Variables are expressed as counts and percentages. The OR and P value were calculated using the chi-square test. HC, healthy controls; OR, odds ratio; pSS, primary Sjögren’s syndrome.

**Supplementary Table S3.** PCR primers

| Gene Name | Forward primer (5'-3') | Reverse primer (5'-3') |
| --- | --- | --- |
| GAPDH | TCAACGACCACTTTGTCAAGCTCA | GCTGGTGGTCCAGGGGTCTTACT |
| GTF2I-γ | ATAACATTCAAGCAGGCCCTT | GGAGAATAATCATCATCTTCTGCTG |
| GTF2I-β | TAACATTCAAGGAAGCCACCA | TCTTCAATAGTCACCTCAACTTCA |
| GTF2I-α | ATAACATTCAAGCAGGCCCTT | CTCAACTTCAGGGTCCTCACT |
| GTF2I-δ | TAACATTCAAGGAAGCCACCA | GGAGAATAATCATCATCTTCTGCTG |
| GTF2I-ε | ACATTCAAGGCCCTTCTGAAAC | GAGAATAATCATCATCTTCTGCTGG |
| GTF2I-ζ | TGGCCCCATCAAAGTGAAAAC | TCTTTTTCTCCTAACCTTCTGCTG |
| TNF-α | CCTCTCTCTAATCAGCCCTCTG | GAGGACCTGGGAGTAGATGAG |
| IL-6 | ACTCACCTCTTCAGAACGAATTG | CCATCTTTGGAAGGTTCAGGTTG |
| IL-1β | ACAGTGGCAATGAGGATG | TGTAGTGGTGGTCGGAGA |
| IFNB | ACTGCAACCTTTCGAAGCCT | AGCCTCCCATTCAATTGCCA |
| c-FOS | CACTCCAAGCGGAGACAGAC | AGGTCATCAGGGATCTTGCAG |
| c-JUN | TGAGTGACCGCGACTTTTCA | TTTCTCTAAGAGCGCACGCA |

**Supplementary Table S4.** c**-**FOS siRNA sequences

|  | sense（5'-3'） | antisense（5'-3'） |
| --- | --- | --- |
| si-FOS 1 | GGGAUAGCCUCUCUUACUATT | UAGUAAGAGAGGCUAUCCCTT |
| si-FOS 2 | CAAGGUGGAACAGUUAUCUTT | AGAUAACUGUUCCACCUUGTT |
