# Supplementary material for: Risk Allele rs117026326-Mediated Alternative Splicing of GTF2I Promotes B Cell Proliferation in Primary Sjögren's Syndrome
Source: J Immunol Res. 2025 Feb 18;2025:4821639. doi: 10.1155/jimr/4821639 (PMC11858827; doi:10.1155/jimr/4821639)
Supplement: Supporting Information 3 — Figure S3: ESE finder prediction of rs117026326 in exon splicing enhancers. [file 4821639.f3.pdf]

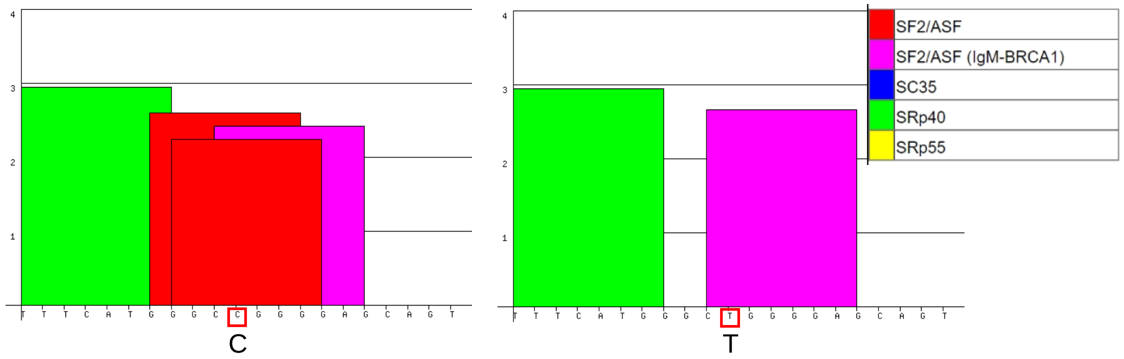
4
3
2
1
T T T C A T G G G C C G G G G A G C A G T
C
4
3
2
1
T T T C A T G G G C T G G G G A G C A G T
T
SF2/ASF
SF2/ASF (IgM-BRCA1)
SC35
SRp40
SRp55
